# Supplementary material for: Controlling nanochannel orientation and dimensions in graphene-based nanofluidic membranes
Source: Nat Commun. 2021 Jan 21;12:507. doi: 10.1038/s41467-020-20837-2 (PMC7820404; doi:10.1038/s41467-020-20837-2)
Supplement: Supplementary file 1 — Supplementary Information [file 41467_2020_20837_MOESM1_ESM.pdf]

Supporting Information

Controlling Nanochannel Orientation and Dimensions in Graphene-Based Nanofluidic Membranes

Muchun Liu[1,2,3], Paula J. Weston[4], Robert H. Hurt[1*]

[1]School of Engineering, Brown University, Providence, RI, USA;

[2]Department of Chemistry, Brown University, Providence, RI, USA;

[3]Department of Civil and Environmental Engineering, Massachusetts Institute of Technology, Cambridge, MA, USA;

[4]Department of Pathology and Laboratory Medicine, Brown University, Providence, RI, USA

* corresponding author email address: robert_hurt@brown.edu

Supplementary Note 1: Synthesis of GO suspensions

Graphite oxide (GO) was synthesized using a modified Hummers' method with a pre-intercalation treatment. Concentrated H_2SO_4 (100 mL) was heated to 80 °C in a 500 mL Erlenmeyer flask. $K_2S_2O_8$ (10 g) and P_2O_5 (10 g) were added to the acid and stirred until fully dissolved. Graphite powder (14 g, Bay Carbon Inc. SP-1 grade) was added to the solution and kept at 80 °C for 5 hrs. The mixture was then cooled and placed in an iced bath, followed with slow dilution by 200 mL deionized (DI) water. The solid sample was filtered using a vacuum filtration unit (47 mm diameter, 0.2 μm pore size), further rinsed with 1 L DI water and then dried in air for overnight. Concentrated H_2SO_4 (500 mL), $NaNO_3$ (10 g) and the pre-oxidized graphite powder were transferred into a 2 L Erlenmeyer flask in an ice bath, then $KMnO_4$ (70 g) was slowly added to the mixture while stirring (temperature was controlled within 10 °C during the complete process). The flask was moved to a 40 °C water bath and left for 3 hrs, and then transferred back into the ice bath. DI water (1 L) was slowly added to the flask while stirring, taking caution that the temperature did not rise above 55 °C. After dilution, 60 mL of a 30% H_2O_2 solution was added to the mixture dropwise. The color of the solution turned from dark brown to bright yellow. This mixture was left under stirring overnight to consume the H_2O_2. The washing process included 5 rounds of acid washing using 1M HCl to remove residual salts (centrifugation at 4000 rpm for 30 min), and 4 rounds of acetone

washing (centrifugation at 4000 for 30 min). After thorough washing, the resulting wet solid was collected and dried in air for 72 hrs. To harvest GO suspensions, approximately 4 g dried sample was fully dispersed in 1 L DI water and bath sonicated for 40 min. The suspension was centrifuged at slow speed (around 1000 rpm) for 5 min, then the supernatant was carefully collected by a pipette. After 3 rounds of centrifugation and supernatant collection, the final GO suspension was obtained for usage. The as-prepared GO nanosheets possess a lateral size approximately 1 μm and thickness approximately 1 nm (Supplementary Fig. 1a). Potential GO impurities N, S, Mn, K, Cl, and P were not detected by XPS. The C1s XPS spectra for GO is shown in Supplementary Fig. 1b, the C/O atomic ratio of GO is approximately 2.1.

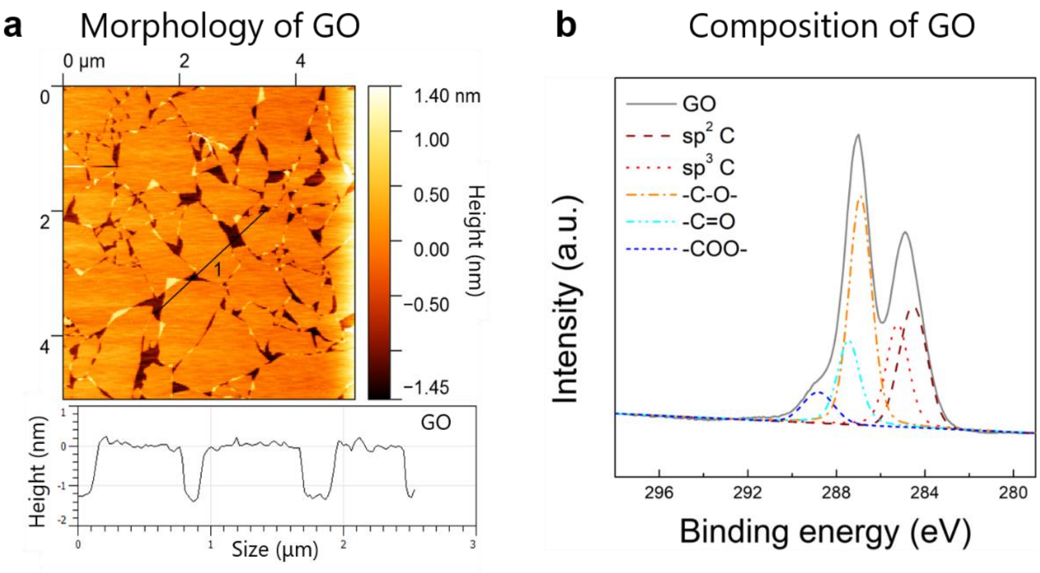

Supplementary Figure 1. Morphology and composition of as-prepared GO nanosheets. a, AFM image and accompanying height profile of GO nanosheets drop-cast from diluted suspension onto mica. The color bar indicates the height of sample. **b**, Surface composition of a GO film. C1s XPS spectra of GO are fitted to five different peaks centered at 284.5, 285.2, 286.9, 287.5 and 288.8 eV, which are associated with sp^2C, sp^3C, C-O, -C=O and -COO- groups, respectively.

Supplementary Note 2: Structures of VAGME films

Fig. 1a and Supplementary Fig. 2 shows idealized and actual structures of the VAGME films in cross section. The top and side views of neat (dopant-free) wrinkled GO films exhibit an irregularly wrinkling pattern instead of the desired regular arrangement of line segments at same height (Supplementary Fig. 2a). Delamination voids are observed between the GO top films and

polystyrene substrates, and these may be responsible for the irregular wrinkle geometries. Delamination is likely due to weak binding between the negatively charged GO nanosheets and the air-plasma-treated polymer substrate. This phenomenon exists in all neat GO samples regardless of the thickness (Supplementary Fig. 2a). The lack of structural regularity makes it difficult to capture the full set of GO wrinkles in any one thin section, resulting in a low yield of open nanochannels.

Supplementary Note 3: Metal cation addition to improve film adhesion and wrinkle uniformity

We attempted to achieve a more regular zig-zag pattern by creating stronger film/substrate adhesion. In our previous report[1], metal cation complexation proved to be effective in preparing colloidally stable, positively charged GO nanosheet suspensions. The binding of metal cations with fully charged acidic sites on GO can compensate for the negative charge, or even reverse the charge at sufficiently high metal-carbon ratio. As shown in Supplementary Fig. 2b, the pure GO aqueous suspension is colloidal stable due to (-/-) repulsion of ionized nanosheets, reflected by a zeta potential of -48 mV at $M^{n+}/C = 0$. Progressing addition of ZrO(II) or Fe(III) cations first reduces the magnitude of the negative charge then induces a charge flip and eventually a second regime of colloidal stability at high positive zeta potential (> +40 or +20 mV) for Zr/C or Fe/C ratios larger than $\frac{1}{22}$. In contrast, other metal cations were observed to cause flocculation at M^{n+}/C ratios greater than approximately $\frac{1.5}{1}$, corresponding to the low-surface-charge window (-15mV < ζ < +15 mV). The C/O atomic ratio of our GO is 2.1, therefore, the C atom molar concentration of our 0.1 mg mL^{-1} GO suspension can be estimated as $(0.1\ \mathrm{mg\ mL^{-1}}) \times [\frac{2.1}{2.1\times 12\ \mathrm{mg\ mmol^{-1}} + 1\times 16\ \mathrm{mg\ mmol^{-1}}} \approx$ 5 mM. The experimental ζ-potential behaviors can be understood through a simplified model of cation complexation[1]. The stability constants $\beta = \frac{[\mathrm{M^{n+}}-\mathrm{GO^*}]}{[\mathrm{M^{n+}}]\times[\mathrm{GO^*}]}$ were estimated using cation-acetate complexes (Supplementary Table 1.), where GO^* represents an oxygen-containing binding site on GO with unit negative charge.

Supplementary Table 1. Standard state stability constant constants for acetate complexes at 25 °C and 1 bar[2-4].

Metal	log β
**ZrO^{2+}**	6.18
**Fe^{3+}**	4.29
**Al^{3+}**	3.02
**Pb^{2+}**	2.70
**Ni^{2+}**	2.12
**Co^{2+}**	1.93
**Cu^{2+}**	2.40
**Ca^{2+}**	1.12

The theoretical surface charge density on M^{n+}-GO nanosheets can be calculated by summation of ionized species on GO nanosheets using equation (1)[5]

$$\sigma = \frac{z_i e c_{iN_A}}{A\rho} = \frac{eN_A(-1\times \mathrm{GO}_{\mathrm{acidic\ sites}} + n_i \times [\mathrm{M}^{\mathrm{n+}} - \mathrm{GO}^*])}{A\rho} \qquad (1)$$

Where z_i is the valency of ith species, c_i is the concentration of bound ith species, $e = 1.6\times10^{-19}$ Coulombs, N_A is Avogadro's constant, A is theoretical specific surface area of GO (estimated as 1609 m^2 g^{-1} by combining theoretical surface area of graphene (2630 m^2 g^{-1}) and atomic C/O ratio of GO approximately 2.1), ρ is mass concentration of GO (0.1 mg mL^{-1}). n_i is effective charge of each M^{n+}-GO binding site, and based on the zeta potential trends of M^{n+}-GO suspensions, we proposed a multi-binding mode for M^{n+}-GO nanosheets, where monoatomic cations (Fe(III, Al(III), Co(II), Ni(II), Pb(II), Cu(II) and Ca(II)) interact with two complexation sites on GO nanosheets, while diatomic cation that contains oxygen, like ZrO(II) interacts with only one complexation site. Therefore, n_i is $\frac{3}{2}$ for Fe(III)-, Al(III)-; $\frac{2}{2}$ for Pb(II)-, Co(II)-, Ni(II)- , Cu(II)-, Ca(II)- and $\frac{2}{1}$ for ZrO(II)-GO colloids.

The Gouy-Chapman equation (2) was used to relate the surface charge σ_s of M^{n+}-GO colloids with their zeta potentials, adopted describing a distribution of dissolved ions at a charged surface (electrical double layer)[5,6],

$$\sigma_s = \frac{2\varepsilon kT\kappa}{ze} \sinh\left(\frac{ze\zeta}{2kT}\right) \qquad (2)$$

where ε is the solution permittivity ($\varepsilon = \varepsilon_r \varepsilon_0$, ε_r = 78.5 is obtained on DLS), k the Boltzman constant, T the temperature, z is the valency of the counter-ions and κ the reciprocal of Debye length (nm^{-1}).

The Debye length is given by the expression $\frac{0.304}{\sqrt{I}}$, where I is the ionic strength defined as $\frac{1}{2}\sum z_i^2[x_i]$; x_i is the molar concentration of the ith species, and z_i is valency. All M^{n+}-GO colloids were prepared in 20 mM $NaNO_3$ aqueous solutions to minimize the effect of different ionic strength on zeta potential (as the conc. of test salts range from 0.05 – 15.00 mM).

By combining equation (1) and (2), the concentration of total acidic sites on GO nanosheets was back-calculated as approximately 0.03 mM (zeta potential of 0.1 mg mL^{-1} GO suspensions is -48 mV). Similarly, by combining equation (1), (2) and the binding stability equilibrium relations, theoretical zeta potentials of M^{n+}-GO were back-calculated at different metal-carbon ratios[5]. As shown in Supplementary Fig. 2b, the theoretical and experimental zeta potentials are in good agreement, indicating the successful surface charge reversion of ZrO(II) and Fe(III) is a synergetic effect of high complexation constant and effective valence as in proposed model. The (+/+) repulsion between Zr- or Fe-GO nanosheets maintains colloidal stability and produces high quality films during subsequent drying induced assembly.

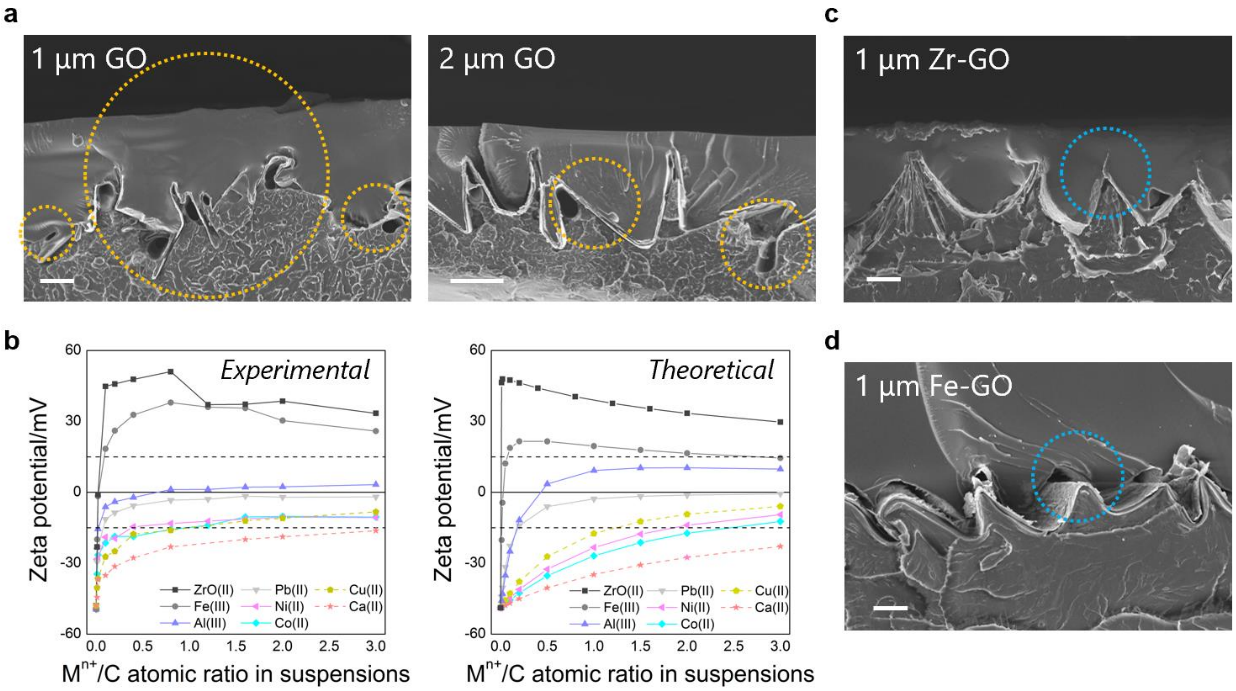

Supplementary Figure 2. Morphologies of wrinkled films and ζ-potential behaviors of metal cation-GO nanosheet suspensions. a, Side (cross-sectional) views of epoxy-fixed neat GO wrinkled films. Left, 1 μm-thick GO films after compressive deformation and embedding in epoxy. Scale bar, 20 μm. Right, 2 μm-thick GO films after compressive deformation and embedding in epoxy. Scale bar, 50 μm. **b**, Experimental and theoretical (model-predicted) ζ-potentials of GO nanosheet suspensions with varying

degrees of metal salt addition, expressed as a function of M^{n+}/C atomic ratio. A series of metal cations are tested, including ZrO(II), Fe(III), Al(III), Ni(II), Co(II), Cu(II) and Ca(II). GO dispersions are all at 0.1 mg mL^{-1} solids loading. Colloidal stability of $ZrOCl_2$ doped GO can be achieved by $\zeta >$ +20mV (+/+ repulsion due to surface charge inversion). **c**, Sides views of metal ion-doped GO/epoxy composites. 1 μm-thick Zr-GO films after compressive deformation and embedding in epoxy. **d**, Sides views of metal ion-doped GO/epoxy composites.1 μm-thick Fe-GO films after compressive deformation and embedding in epoxy. Scale bar, 20 μm.

Multivalent metal cations ZrO(II) and Fe(III) were doped into the films by depositing GO from $ZrOCl_2$ or $Fe(NO_3)_3$ solutions to introduce (+/-) electrostatic attraction at the GO-substrate interface. The side views of Zr- and Fe-GO wrinkled films are shown in Supplementary Fig. 2c-d. Fe- and Zr-doped GO wrinkled films possess a more regular zig-zag pattern. Furthermore, the Zr-GO wrinkled films (Supplementary Fig. 2c) exhibit higher ridge curvature (sharper corners) and stronger affinity (no detachment voids) than the Fe-GO films (Supplementary Fig. 2d), which may be due to the higher zeta potential on Zr- (+40 mV) than Fe-GO (+20 mV) nanosheets at same metal-carbon loading ($M^{n+}/C = \frac{1}{22}$), which maximizes the doping effect and facilitates the subsequent sectioning. The further development of VAGMEs was therefore pursued using Zr-doped GO.

The microtome sectioning technique plays an important role in obtaining an intact VAGME thin film. Several potential film flaws are shown in Supplementary Fig. 3. For very thin sections (cut thickness < 10 μm), the 1 μm Zr-GO strips may fragment during sectioning (Supplementary Fig. 3a). Use of very thick Zr-GO strips (> 2 μm) can lead to internal delamination within the strips after sectioning (Supplementary Fig. 3b). In Supplementary Fig. 3c, multiple holes are observed in the VAGME, which originated as trapped air bubbles in the epoxy caused by insufficient degassing. Finally, full curing of the epoxy at 60 °C makes the matrix quite stiff and can produce interfacial delamination between Zr-GO films and resin during sectioning (Supplementary Fig. 3d). Observing and then avoiding these structural defects led to the final protocol for VAGME fabrication (see Methods).

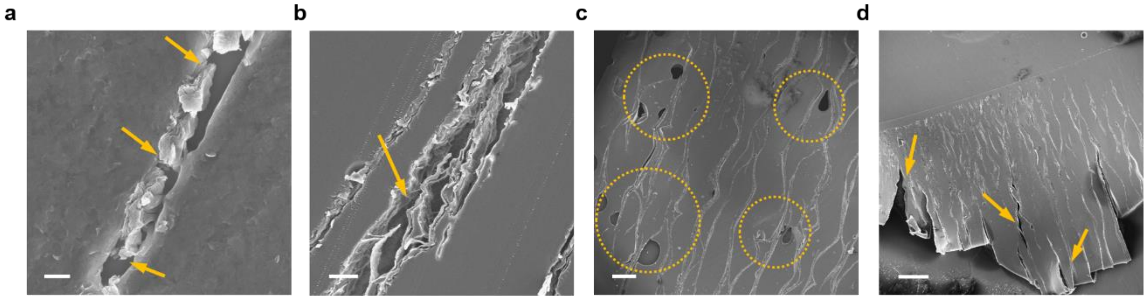

Supplementary Figure 3. Structural failures of VAGME appeared in developing microtome sectioning technique. **a**, Zr-GO films (Zr/C = $\frac{1}{22}$) fragment at cut thickness of 2 μm. Scale bar, 2 μm. **b**, Delamination of nanosheets at Zr-GO precursor (Zr/C = $\frac{1}{60}$) planar film thickness of 2 μm. Scale bar, 10 μm. **c**, Holes on VAGME caused by air bubbles after a 5-min degassing period. Scale bar, 100 μm. **d**, Delamination at the interfaces of Zr-GO films and epoxy while epoxy is fully cured at 60 °C. Scale bar, 100 μm.

As shown in Supplementary Fig. 4a and 1a, the GO nanosheets are approximately 1 μm in lateral size and 1 nm in thickness, with a relatively broad distribution in lateral dimension, as is typical of exfoliated materials. During drying induced assembly, GO nanosheets align and stack by volume exclusion effects into an irregular tiling pattern to form a paper-like macroscopic structure (Supplementary Fig. 4b-c) where nanosheets are held together by hydrogen bonding, van der Waals forces, and π-π interactions in unfunctionalized regions, providing strong adhesion within the layers of the lamellar paper-like structure[7-9]. Such GO films therefore possess significant mechanical strength, which enables the fabrication of various GO multilayered structures, including wrinkled and crumpled films, and hierarchical deformed topographies[10-12]. The cross section of a GO planar film in Supplementary Fig. 4c exhibits well-ordered layer packets, in which each packet consists of approximately 7 stacked GO nanosheets[7]. The ordered packets of nanosheets in GO films remain during deformation and realignment (Supplementary Fig. 4d).

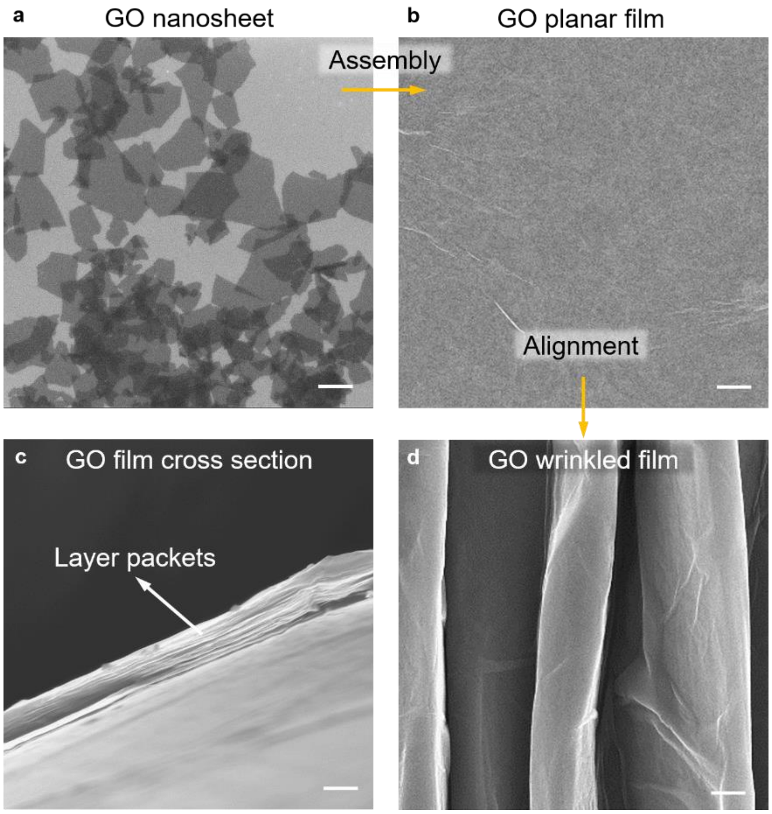

Supplementary Figure 4. Assembly and alignment of GO nanosheets. **a**, Top view of individual GO nanosheets imaged by drop casting GO dilute suspension on a silicon substrate and SEM. **b**, Top view of a planar GO film assembled from GO nanosheets, which are smooth except for fine wrinkle structures that form spontaneously upon drying. **c**, Cross-sectional view of 1 μm thick GO planar film. **d**, Top view of a wrinkled GO film by unidirectional compressions of a planar GO film, showing the smooth continuous nature of the film surface. Scale bar, 1 μm.

Similarly, casting Zr-GO nanosheet suspensions onto substrates produces intercalated laminated films as shown by time-resolved XRD in Supplementary Fig. 5a. During the drying process, the Zr-GO nanosheets slowly stack together and form a lamellar structure, exhibiting an interlayer spacing of 8.8 angstroms. After realignment into VAGME, the GO strips still exhibit multi-layered structures as shown in Supplementary Fig. 5b. From a fractured VAGME (Supplementary Fig. 5c) we can see the tilted Zr-GO strips attached on the side of the epoxy, and the fine, irregular kinks in the film (in the X direction in Fig. 5b) appear now as near-vertical ridges in the fracture surface (Fig. 5c), which also reflect the near-vertical alignment of the nanochannels within the portion of the film strip attached to the epoxy.

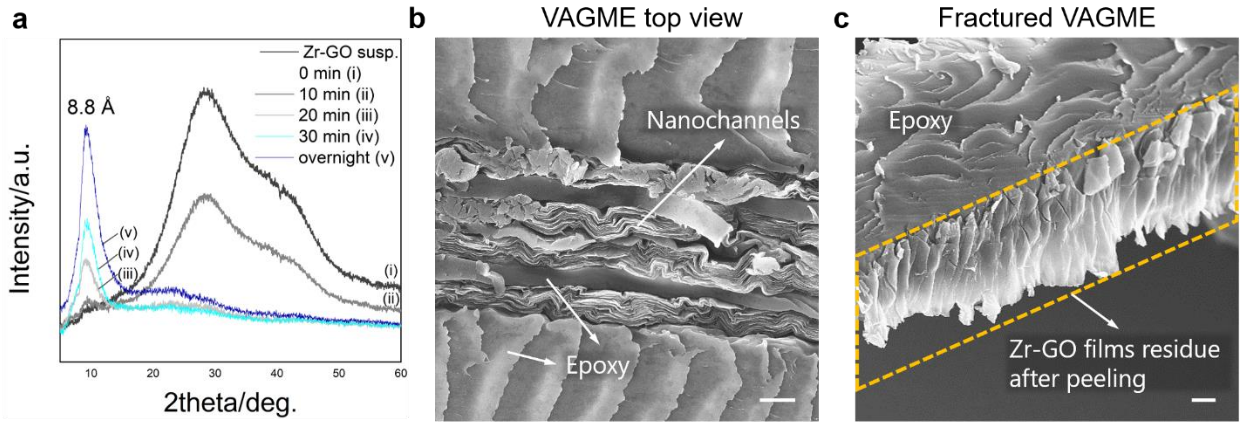

Supplementary Figure 5. Assembly of Zr-GO nanosheets in VAGME. a, Time-resolved XRD tracking the appearance of lamellar structures in Zr-GO films during drying, including Zr-GO suspensions after 0 (i), 10 (ii), 20 (iii), 30 min (iv) and overnight (v) drying. **b**, Top view of Zr-GO strips on the VAGME surface, exhibiting multi-layered structure. **c**, Side view of a fractured VAGME, exposing the exfoliated Zr-GO short films attached on the side of epoxy matrix. Scale bar, 2 μm.

Supplementary Note 4: Nanochannel transport in GO and VAGME

In general, GO possesses oxidized and non-oxidized regions, where oxygen-containing groups function as spacers between nanosheets. Water molecules enter nanochannels driven by capillary forces, and are reported to experience frictionless flow through non-oxidized regions or slower flow though oxidized regions[13,14]. The size of the non-oxidized channels can be estimated by subtracting the width of a pristine (non-oxidized) graphene layer (0.34 nm) from the XRD interlayer spacing (here 0.88 nm) to give 0.54 nm. In some materials with high oxygen content, the oxidized regions form the continuous network, leaving non-oxidized regions as isolated islands[15,16]. In these cases, molecular transport in GO membranes may be forced to occur primarily through the oxidized regions. The sizes of those channels can be estimated here as 0.88 nm (measured d-spacing of Zr-GO films) – 0.34 nm (graphene layer thickness) – 0.2 nm (functional group size) = 0.34 nm, for alternative estimate of the relevant channel size for molecular exclusion phenomena in this study[17-19].

Supplementary Table 2 shows the fluxes of water and 2-proposal vapor above their liquid mixtures. The water/2-propanol system is highly non-ideal and azeotropic, but vapor/liquid equilibrium data are widely available, and the component vapor pressures (partial pressures) above the liquid mixtures are also given in Supplementary Table 2[20]. The VAGME selectivity for water over 2-propanol is above a factor of 100 times. The corresponding calculation process is shown in Supplementary Fig. 6 and Table 3.

Supplementary Table 2 Water and 2-Propanol vapor fluxes of liquid mixtures measured through VAGME devices.

**Component in the feed liquid mixtures**	**Flux (80 °C)** mmol mm^{-2} hr^{-1}	**Vapor pressure above non-ideal liquid mixtures*** bar
**Water**	5.0±0.2	0.45
**2-Propanol**	(6.2±3.7) $\times 10^{-2}$	0.55

*Vapor pressures are estimated from vapor-liquid equilibrium plot for water-2-propanol[20].

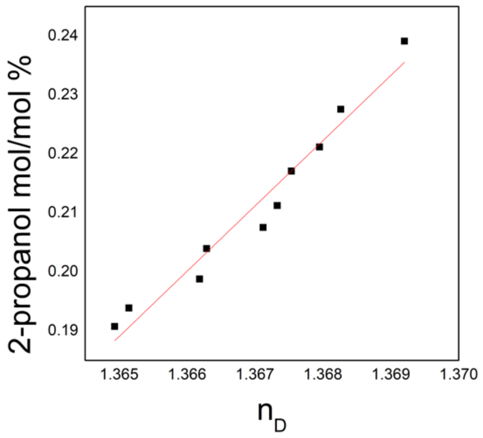

Supplementary Figure 6. Calibration curve of refractive index (n_D) and 2-propanol molar ratio in the liquid mixtures.

Supplementary Table 3. Calculation parameters for water/2-propanol liquid mixtures.

**Mass loss of liquid mixtures (mg hr^{-1})**	84.1±4.9
$\boldsymbol{n_D}$ **(before)**	1.3649±0.001

**IPA mol% (before)**	19.1±0.1%
$\boldsymbol{n}_{\mathbf{D}}$ **(after)**	1.3655±0.001
**IPA mol% (after)**	19.4±0.1%

The water vapor flux through VAGME at 100 °C is shown in Supplementary Fig. 7a, and shows a gradual decline in water flux over time. We hypothesized this decline is due to the gradual thermal reduction (deoxygenation) of GO which results in decrease of chemical polarity and affinity for water[21,22]. Interestingly, XRD results does not show a significant change of interlayer spacing in Zr-GO films upon 100 °C water vapor treatment. The interlayer spacing remain at 8.8 angstrom (Supplementary Fig. 7b), which may be due to the presence of ZrO(II) ions that continue to act as pillars to set the spacing even while oxygen functional groups are removed[13]. XPS tests were therefore carried out to probe the chemical transformation of GO nanosheets during thermal treatment. Since Zr-GO films contain ZrO(II) and absorbed water molecules, the cumulative O/C ratio cannot be used to reliably monitor the oxygen content of GO nanosheets. Instead we used the atomic ratio of oxidized carbon to total carbon to track the degree of thermal reduction. From the trend in Supplementary Fig. 7c we can see the oxygen content declines significantly after 12 hrs of exposure, which is consistent with permeation results. The data are calculated from fitting profiles of C1s peaks as shown in Supplementary Fig. 8. Therefore, thermal reduction of GO nanosheets results in a decline in hydrophilicity, which further supports that molecules can only transport through Zr-GO nanochannels on VAGME.

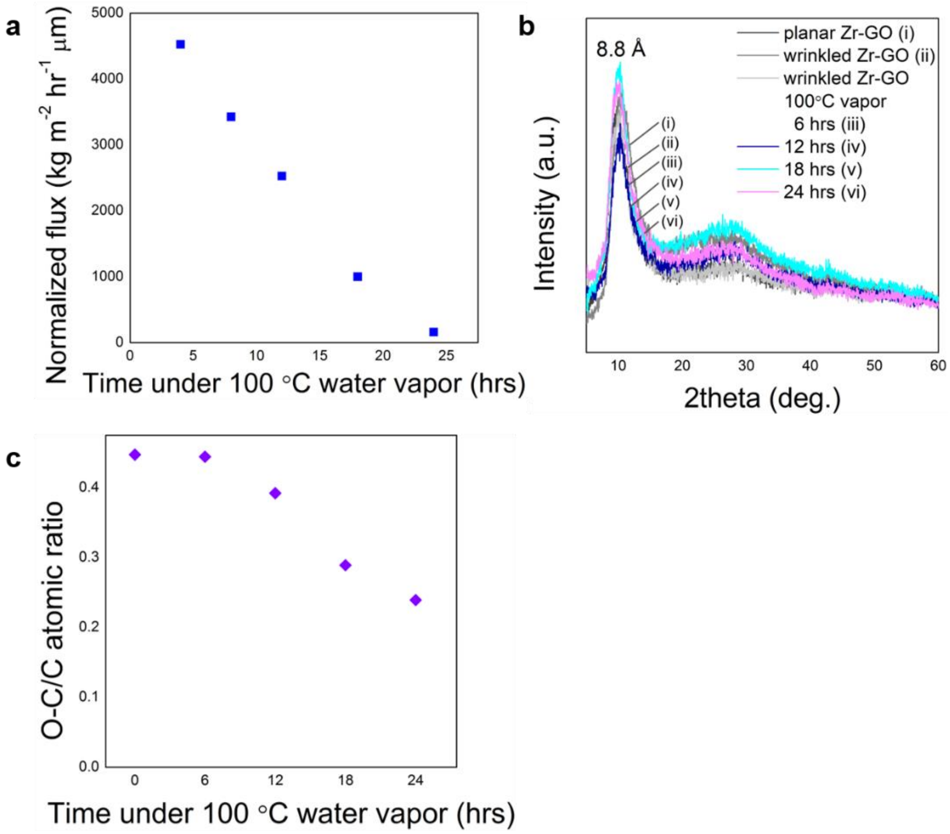

Supplementary Figure 7. Time-dependent behavior and properties of VAGMEs during exposure to water vapor at 100 °C. a, Time-resolved measurements of water vapor transmission fluxes at 100 °C over the course of 24 hrs exposure to the 100 °C water vapor permeate. **b**, XRD spectra of Zr-GO films after different treatments, including planar (i), thermally activated compressive wrinkling (ii) and 100 °C water vapor exposure for 6 (iii), 12 (iv), 18 (v) and 24 hrs (vi). **c**, O-C/C atomic ratios by XPS for wrinkled Zr-GO films after 100 °C water vapor treatment for 0, 6, 12, 18 and 24 hrs for monitoring deoxygenation.

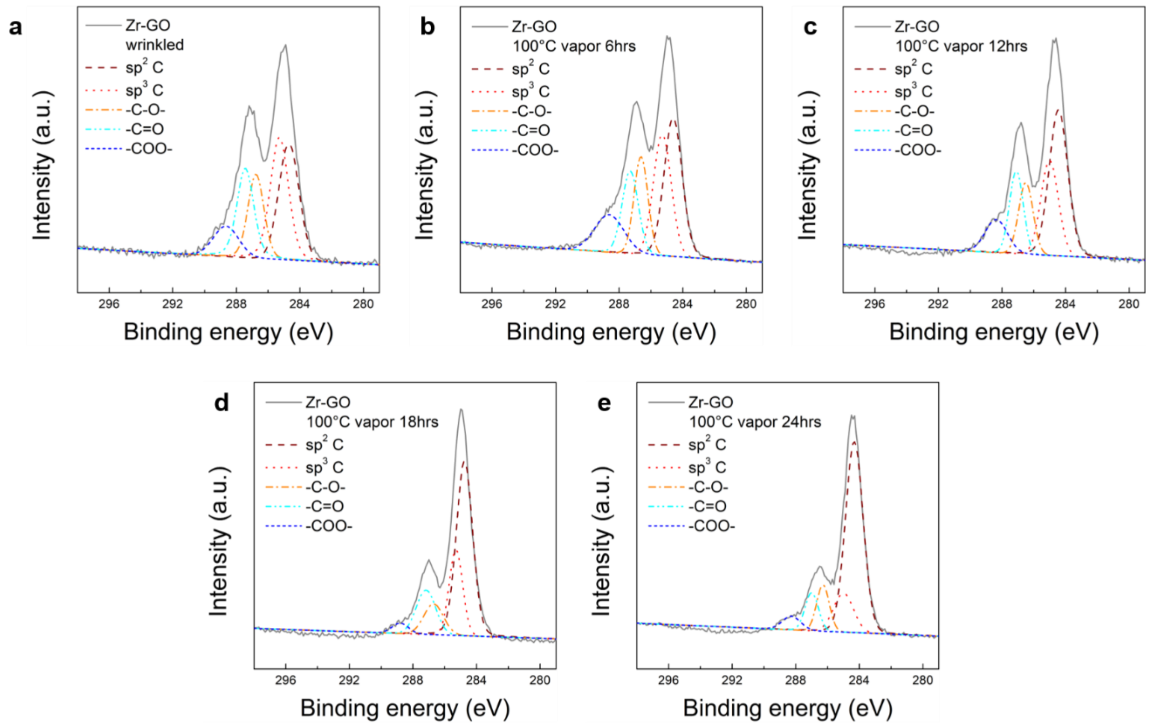

Supplementary Figure 8. C1s XPS spectra for wrinkled Zr-GO films to 100 °C water vapor for various times, in hrs: (**a**) 0, (**b**) 6, (**c**) 12, (**d**) 18, (**e**) 24. C1s XPS spectra of GO are fitted to five different peaks centered at 284.5, 285.2, 286.9, 287.5 and 288.8 eV, which are associated with sp^2C, sp^3C, C-O, -C=O and -COO- groups, respectively.

All liquid filtration tests were done using the device shown in Supplementary Fig. 9. Permeation data for NaCl and $MgCl_2$ aqueous solutions through VAGME are shown in Supplementary Fig. 10 and Supplementary Table 4. The permeation rate of NaCl is two orders of magnitude higher than that of $MgCl_2$, consistent with data from Abraham, J. et al[23]. The selectivity for Na^+ is likely due to its smaller hydrated diameter and/or its larger crystal radius and smaller charge, which weakens the hydration shell, making it easier to partially detach when entering or transporting through nanochannels[24-26].

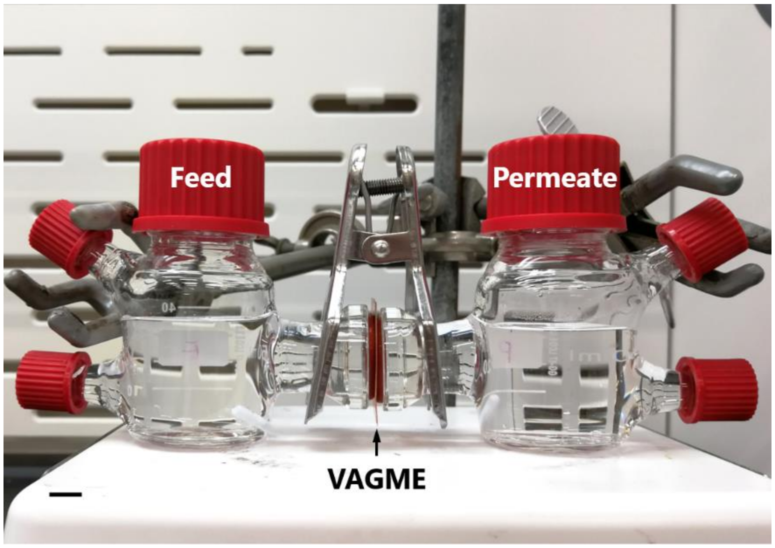

Supplementary Figure 9. Liquid filtration device. Experimental set-up showing H-shaped glass cells as feed and permeate compartments. Scale bar, 1 cm. VAGME was clamped between two O-rings and then fixed between feed and permeate compartments to provide a leak tight environment for the liquid filtration experiments.

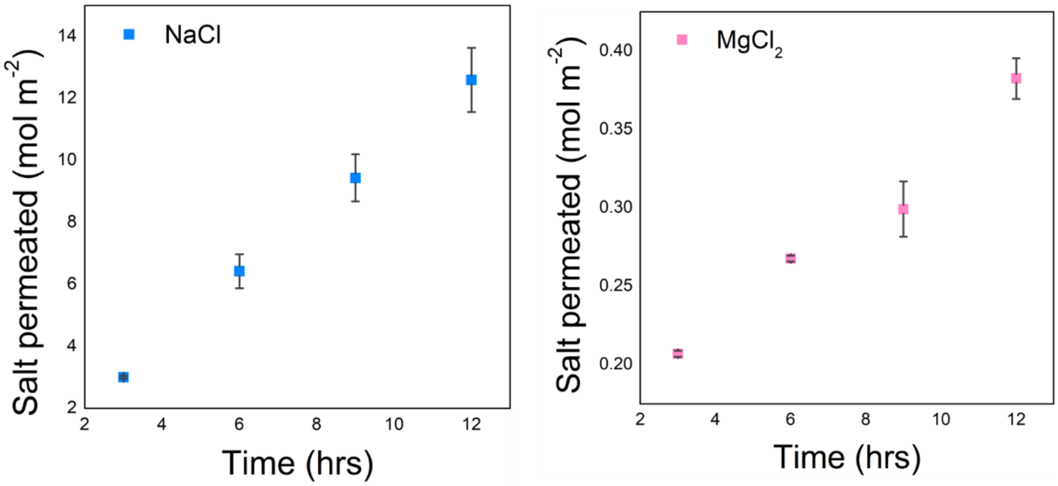

Supplementary Figure 10. Salts permeation through VAGME. Permeation through a VAGME membrane from the feed compartment with 0.05 M aqueous solution of NaCl or $MgCl_2$.

Supplementary Table 4. Permeation rates of ions through VAGME and the hydrated diameter of metal ions.

Ions	**Permeation rate** mol m^{-2} hr^{-1}	**Hydrated diameter**[24,26] nm

**Na(I)**	1.1±0.1	0.45-0.72
**Mg(II)**	(1.9±0.2) $\times 10^{-2}$	0.60-0.94

Supplementary References

1. Liu, M., Chen, P.-Y. & Hurt, R. H. Graphene inks as versatile templates for printing tiled metal oxide crystalline films. *Adv. Mater.* **30**, 1705080 (2018).
2. Shock, E. L. & Koretsky, C. M. Metal-organic complexes in geochemical processes: Estimation of standard partial molal thermodynamic properties of aqueous complexes between metal cations and monovalent organic acid ligands at high pressures and temperatures. *Geochim. Cosmochim. Acta* **59**, 1497-1532 (1995).
3. Perrin, D. D. & Sillén, L. G. *Stability constants of metal-ion complexes, part B : organic ligands*. (Pergamon Press, 1979).
4. Sillén, L. G., Martell, A. E. & Bjerrum, J. *Stability constants of metal-ion complexes*. (Chemical Society, 1964).
5. Konkena, B. & Vasudevan, S. Understanding aqueous dispersibility of graphene oxide and reduced graphene oxide through pKa measurements. *J. Phys. Chem. Lett.* **3**, 867-872 (2012).
6. Jimbo, T., Higa, M., Minoura, N. & Tanioka, A. Surface characterization of poly(acrylonitrile) membranes graft-polymerized with ionic monomers as revealed by ζ potential measurement. *Macromolecules* **31**, 1277-1284 (1998).
7. Dikin, D. A. *et al.* Preparation and characterization of graphene oxide paper. *Nature* **448**, 457-460 (2007).
8. Medhekar, N. V., Ramasubramaniam, A., Ruoff, R. S. & Shenoy, V. B. Hydrogen bond networks in graphene oxide composite paper: structure and mechanical properties. *ACS Nano* **4**, 2300-2306 (2010).
9. Compton, O. C., Dikin, D. A., Putz, K. W., Brinson, L. C. & Nguyen, S. T. Electrically conductive "alkylated" graphene paper via chemical reduction of amine-functionalized graphene oxide paper. *Adv. Mater.* **22**, 892-896 (2010).
10. Chen, P.-Y., Liu, M., Wang, Z., Hurt, R. H. & Wong, I. Y. From flatland to spaceland: higher dimensional patterning with two-dimensional materials. *Adv. Mater.* **29**, 1605096 (2017).
11. Chen, P.-Y. *et al.* Multiscale graphene topographies programmed by sequential mechanical deformation. *Adv. Mater.* **28**, 3564-3571 (2016).
12. Chen, Y. *et al.* Aerosol synthesis of cargo-filled graphene nanosacks. *Nano Lett.* **12**, 1996-2002 (2012).
13. Mi, B. Graphene oxide membranes for ionic and molecular sieving. *Science* **343**, 740-742 (2014).
14. Joshi, R. K. *et al.* Precise and ultrafast molecular sieving through graphene oxide membranes. *Science* **343**, 752-754 (2014).
15. Erickson, K. *et al.* Determination of the local chemical structure of graphene oxide and reduced graphene oxide. *Adv. Mater.* **22**, 4467-4472 (2010).
16. Pacilé, D. *et al.* Electronic properties and atomic structure of graphene oxide membranes. *Carbon* **49**, 966-972 (2011).

17. Zheng, S., Tu, Q., Urban, J. J., Li, S. & Mi, B. Swelling of graphene oxide membranes in aqueous solution: characterization of interlayer spacing and insight into water transport mechanisms. *ACS Nano* **11**, 6440-6450 (2017).
18. Schniepp, H. C. *et al.* Functionalized single graphene sheets derived from splitting graphite oxide. *J. Phys. Chem. B* **110**, 8535-8539 (2006).
19. Lecaros, R. L. G. *et al.* Tunable interlayer spacing of composite graphene oxide-framework membrane for acetic acid dehydration. *Carbon* **123**, 660-667 (2017).
20. Lebo, R. B. Properties of mixtures of isopropyl alcohol and water *J. Am. Chem. Soc.* **43**, 1005-1011 (1921).
21. Pei, S. & Cheng, H.-M. The reduction of graphene oxide. *Carbon* **50**, 3210-3228 (2012).
22. Dreyer, D. R., Park, S., Bielawski, C. W. & Ruoff, R. S. The chemistry of graphene oxide. *Chem. Soc. Rev.* **39**, 228-240 (2010).
23. Abraham, J. *et al.* Tunable sieving of ions using graphene oxide membranes. *Nat. Nanotechnol.* **12**, 546-550 (2017).
24. Kielland, J. Individual activity coefficients of ions in aqueous solutions. *J. Am. Chem. Soc.* **59**, 1675-1678 (1937).
25. Tansel, B. *et al.* Significance of hydrated radius and hydration shells on ionic permeability during nanofiltration in dead end and cross flow modes. *Sep. Purif. Technol.* **51**, 40-47 (2006).
26. Tansel, B. Significance of thermodynamic and physical characteristics on permeation of ions during membrane separation: Hydrated radius, hydration free energy and viscous effects. *Sep. Purif. Technol.* **86**, 119-126 (2012).
